# Polyphenol Iongel Patches with Antimicrobial, Antioxidant and Anti-Inflammatory Properties

**DOI:** 10.3390/polym15051076

**Published:** 2023-02-21

**Authors:** Gisela C. Luque, Melissa Moya, Matias L. Picchio, Vanessa Bagnarello, Idalia Valerio, José Bolaños, María Vethencourt, Sue-Hellen Gamboa, Liliana C. Tomé, Roque J. Minari, David Mecerreyes

**Affiliations:** 1 Instituto de Desarrollo Tecnológico para la Industria Química (INTEC), CONICET, Güemes 3450, Santa Fe 3000, Argentina; 2 Laboratorio de Investigación, Universidad de Ciencias Médicas, San José 10108, Costa Rica; 3 Facultad de Microbiología, Universidad de Ciencias Médicas, San José 10108, Costa Rica; 4 POLYMAT, University of the Basque Country UPV/EHU, Joxe Mari Korta Center, Avda. Tolosa 72, 20018 Donostia-San Sebastian, Spain; 5 Escuela de Fisioterapia, Universidad de Ciencias Médicas, San José 10108, Costa Rica; 6 Facultad de Medicina, Universidad de Ciencias Médicas, San José 10108, Costa Rica; 7 LAQV-REQUIMTE, Chemistry Department, NOVA School of Science and Technology, FCT NOVA, Universidade NOVA de Lisboa, 2829-516 Caparica, Portugal; 8 Facultad de Ingeniería Química, Universidad Nacional del Litoral, Santiago del Estero 2829, Santa Fe 3000, Argentina; 9 Ikerbasque, Basque Foundation for Science, 48013 Bilbao, Spain

**Keywords:** iongels, biocompatible ionic liquids, wound healing

## Abstract

There is an actual need for developing materials for wound healing applications with anti-inflammatory, antioxidant, or antibacterial properties in order to improve the healing performance. In this work, we report the preparation and characterization of soft and bioactive iongel materials for patches, based on polymeric poly(vinyl alcohol) (PVA) and four ionic liquids containing the cholinium cation and different phenolic acid anions, namely cholinium salicylate ([Ch][Sal]), cholinium gallate ([Ch][Ga]), cholinium vanillate ([Ch][Van]), and cholinium caffeate ([Ch][Caff]). Within the iongels, the phenolic motif in the ionic liquids plays a dual role, acting as a PVA crosslinker and a bioactive compound. The obtained iongels are flexible, elastic, ionic conducting, and thermoreversible materials. Moreover, the iongels demonstrated high biocompatibility, non-hemolytic activity, and non-agglutination in mice blood, which are key-sought material specifications in wound healing applications. All the iongels have shown antibacterial properties, being PVA-[Ch][Sal], the one with higher inhibition halo for *Escherichia Coli*. The iongels also revealed high values of antioxidant activity due to the presence of the polyphenol, with the PVA-[Ch][Van] iongel having the highest activity. Finally, the iongels show a decrease in NO production in LPS-stimulated macrophages, with the PVA-[Ch][Sal] iongel displaying the best anti-inflammatory activity (>63% at 200 µg/mL).

## 1. Introduction

Nowadays, bioactive natural compounds are gaining interest in the biomedical field because of the possibility to obtain biocompatible, biodegradable, and non-toxic materials with therapeutic properties [1,2]. In particular, the design of original skin healthcare materials, like wound healing membranes, is increasing significantly [3,4]. The wound healing mechanism basically involves four steps: hemostasis, inflammation, proliferation, and remodeling [5]. For this reason, evaluating the anti-inflammatory and antioxidant activity of substances with potentiality for wound treatment is crucial because the upregulation of inflammatory mediators and radical oxygen species (ROS) may delay and impair the healing process. Indeed, it is reported that dysregulation of the inflammatory response may result in host tissue damage, rendering a chronic pathological inflammation [6,7]. At the same time, the presence of free radicals and oxidative reactions can accelerate inflammation, and oxidative stress can induce cellular damage, which are the main causes of delayed wound healing.

The use of bioactive materials with anti-inflammatory and antioxidant properties can offer an excellent opportunity to design novel patches for wound treatment and tissue regeneration [6,7]. In this context, polyphenols appear as good candidates because of their attractive properties like analgesic, anti-inflammatory [8], and antioxidant agents [9,10]. Moreover, polyphenols have acted as crosslinkers of different polymers due to their capability to form physical and chemical bindings [11,12]. There are some works reporting the use of phenolic compounds (PhCs) in the synthesis of hydrogels and iongels [11,13,14,15]. Iongels, in particular, are a new generation of soft-ionic materials that can be applied in several fields, such as drug delivery [16,17,18], sensors [14,19], energy [20,21,22], and bioelectronics [23,24]. These exciting materials are tridimensional polymeric networks that contain percolated an ionic liquid (IL) in their structure [25]. For bio-related applications, the polymer and ILs employed need to be biocompatible, biodegradable, and preferably bioactive.

Despite the valuable features of iongels, there are scarce reports regarding their use in wound healing applications, but ILs have been widely combined with biopolymers to develop new materials for this application. Morais et al. reported the preparation of bacterial nanocellulose membranes impregnated with cholinium-based phenolic ionic liquids for skin treatment [26]. They demonstrated by in vitro assays that these membranes present anti-inflammatory and analgesic properties due to the presence of phenolic acid anions in the ILs’ composition. Nevertheless, the main drawback of these materials is their rigidity and low deformation capability (reported elongation at break was around 2%), limiting the surface adaptability during application. In the same line Arruda Fernandez et al. summarizes the advantages to combine the properties of bacterian cellulose with phenolic compounds to prevent the UV-induced skin damage [27]. Moreover, particularly employing cholinium gallate in combination with silk fibroin Gomez et al. reported the preparation of sponges with antioxidant and anti-inflammatory features to be in tissue engineering strategies due to the benefits of the phenolic compound. They demonstrate the influence of the use of the PhCs to balance the pro- and anti-inflammatory cytokines and also no hemolysis effect was informed [28]. Taking advantage of the properties of polyphelons, Shengye and co-workers reported the use of poly(tannic acid) nanorods in a polysaccharide matrix composed by quaternary ammonium chitosan and oxidized β-glucan in the design of hydrogels for diabetic wound healing [29]. They reported of superior wound repair properties of the hydrogel by using diabetic rat model in comparison with a commercial wound dressing.

On the other hand, Fang et al. took advantage of the antibacterial properties of imidazolium-based ILs to synthesize hydrogels from 1-vinyl-3-butylimidazolium bromide poly(ionic liquid) and polyvinyl alcohol (PVA), finding that these obtained materials promoted epidermis reconstruction [30].

In this article, we present, with a different approach using iongels as soft ionic materials, the preparation of bioactive and soft-ionic materials bearing a biocompatible PVA network and percolated phenolic-based ILs. First, four different ILs were synthesized, by combining the cholinium cation and phenolic acids as anions, namely gallate, vanillate, caffeate, and salicylate. The ILs play a dual role in these featured iongels: (*i*) as a physical crosslinker by forming H-bonding with PVA and (*ii*) as bioactive compounds. The mechanical properties of the iongels were fully investigated depending on the material composition, looking for soft, flexible and elastic materials. Furthermore, the potential of the prepared iongel materials for wound healing was studied in terms of their biocompatibility, non-hemolytic properties, and antioxidant and anti-inflammatory activity.

## 2. Experimental

### 2.1. Materials

Poly (vinyl alcohol) (PVA, Merck (Rahway, NJ, USA), degree of hydrolysis 99%, Mw 145 kDa), gallic acid (GA, Merck, ≥99.0%), salicylic acid (SA, Alfa Aesar, Haverhill, MA, USA), caffeic acid (Cam TCI), vanillic acid (VA, Sigma Aldrich, St. Louis, MO, USA), choline hydroxide solution (Sigma Aldrich) were used as supplied. Distilled-deionized water was used for all experiments. The following reagents were used as received: RPMI 1640 (Thermofisher, Waltham, MA, USA), fetal bovine serum (Sigma Aldrich), PSN (penicillin, streptomycin, neomycin) (Gibco), Trypsin (MP biomedics). Trypan blue (Gibco), CyQuant XTT cell Viability assay (Invitrogen), phosphate buffer saline (Sigma Aldrich). 2,2-diphenyl-1-1picrylhydrazyl (Sigma Aldrich), ascorbic acid (Sigma Aldrich), methanol (Sigma Aldrich), lipopolysaccharide (LPS, Sigma Aldrich), N- 1-(naphthyl) ethylenediamine dihydrochloride (Sigma Aldrich), sulfanilamide (Sigma Aldrich), phosphoric acid (Sigma Aldrich), nitrite standard for IC (Sigma Aldrich), ethanol (Sigma Aldrich) and parthenolide (Sigma Aldrich). 

### 2.2. Synthesis of Phenolic-Based Ionic Liquids

The ILs, namely cholinium salicylate ([Ch][Sal]), cholinium gallate ([Ch][Ga]), cholinium vanillate ([Ch][Van]), and cholinium caffeate ([Ch][Caff]) were synthesized using a procedure previously reported by Sintra et al. [31] The chemical structures and purities of the ILs were confirmed by ^1^H- and ^13^C-MR.

*Cholinium gallate:* ^1^H NMR (D_2_O, 400 MHz): *δ*/ppm = 6.98 (s, 2H, H-2 and H-6), 3.97–3.92 (m, 2H, NCH_2_CH_2_OH), 3.39–3.36 (m, 2H, NCH_2_CH_2_OH), 3.09 (s, 9H, N(CH_3_)_3_).

^13^C NMR (D_2_O, 101 MHz): *δ*/ppm = 174.83 (COO), 144.33 (C-3 and C-5), 135.72 (C-4), 128.19 (C-1), 109.33 (C-2 and C-6), 67.43 (t, *J_CN_* = 2.9 Hz, NCH_2_CH_2_OH), 55.54 (NCH_2_CH_2_OH), 53.57 (t, *J_CN_* = 3.9 Hz, N(CH_3_)_3_).

*Cholinium vanillate:* ^1^H NMR (D_2_O, 400 MHz): *δ*/ppm = 7.46 (d, 1H, H-2), 7.39 (dd, 1H, and H-6), 7.36 (d, 1H, H-5), 4.00- 3.95 (m, 2H, NCH_2_CH_2_OH), 3.84 (s, 3H, OCH_3_), 3.45–3.42 (m, 2H, NCH_2_CH_2_OH), 3.12 (s, 9H, N(CH_3_)_3_).

^13^C NMR (D_2_O, 101 MHz): *δ*/ppm = 175.02 (COO), 148.17 (COH-4), 146.78 (COCH_3_-3), 128.40 (CCOO^−1^), 123.08 (C-6), 114.81 (C-5), 113.04 (C-2), 67.30 (t, NCH_2_CH_2_OH), 55.77 (NCH_2_CH_2_OH), 55.51 (OCH3), 53.7, (t, N(CH_3_)_3_).

*Cholinium salicylate:* ^1^H NMR (D_2_O, 400 MHz): *δ*/ppm = 7.65 (d, 1H, H-6), 7.24 (t, 1H, H-4), 6.75 (m, 2H, H-3 and H-5), 3.80–3.75 (m, 2H, NCH_2_CH_2_OH), 3.20–3.17 (m, 2H, NCH_2_CH_2_OH), 2.89 (s, 9H, (N(CH_3_)_3_).

^13^C NMR (D_2_O, 101 MHz): *δ*/ppm = 175.02 (COO), 159.63 (COH-2), 134.02 (C-4), 130.46 (C-6), 119.44 (CCOO^−1^), 117.81 (C-5), 116.32 (C-3), 67.23 (t, JCN = 3.1 Hz, NCH2CH2OH), 55.56 (NCH2CH2OH), 53.66 (t, JCN = 3.9 Hz, N(CH3)3).

*Cholinium caffeate:* ^1^H NMR (*d_6_*-DMSO, 400 MHz): *δ*/ppm = 7.06 (d, 1H, CHCHCOO), 6.87 (d, 1H, H-2), 6.73 (dd, 1H, H-6), 6.66 (d, 1H, H-5), 6.09 (d, 1H, CHCHCOO), 3.87–3.82 (m, 2H, NCH_2_CH_2_OH), 3.43–3.39 (m, 2H, NCH_2_CH_2_OH), 3.11 (s, 9H, (N(CH_3_)_3_)

^13^C NMR (*d_6_*-DMSO, 101 MHz): *δ*/ppm = 171.55 (COO), 148.14 (CHCHCOO), 146.54 (COH-4), 138.27 (COH-3), 126.75 (CHCHCOO), 123.43 (C-1), 119.76 (C-6), 119.21 (C-5), 116.49 (C-2), 67.05 (t, *J_CN_* = 2.8 Hz, NCH_2_CH_2_OH), 55.05 (NCH_2_CH_2_OH), 53.14 (t, *J_CN_* = 3.8 Hz, N(CH_3_)_3_.

### 2.3. Preparation of Self-Assembly Iongel Materials

The iongel materials were formed by hydrogen bonding between PVA and the different phenolic anions of ILs. Iongels with 5, 10, and 20% of polymer concentration (with respect to the IL) were synthesized. The synthesis involved the dissolution of the PVA in water at 90 °C and the addition of the corresponding IL with a ratio water/IL 1:1. For example, to prepare the PVA-[Ch][Sal] iongel with 10 wt% of polymer concentration, 0.05 g of PVA was first dissolved at 90 °C in 0.5 g of water under vigorous stirring. Then, 0.5 g of [Ch][Sal] IL was added. After complete dissolution of all the components, the mixed solution was poured into silicone molds and left at room temperature until gelation.

## 3. Characterization of Iongel Materials

### 3.1. Thermal Analysis

Thermogravimetric analyses (TGA) were carried out on a TGA Q500 device from TA instruments. Samples of around 10 mg were heated at a constant rate of 10 °C min^−1^, under a nitrogen atmosphere, from 25 to 600 °C. The temperature at the maximal decomposition rate (T_max_) was determined at the main peak of the derivative weight loss curve.

### 3.2. FTIR Spectroscopy

A Bruker ALPHA spectrometer was used to collect the attenuated total reflection Fourier transform infrared (ATR-FTIR) spectra, from 400 to 4000 cm^−1^, with a resolution of 4 cm^−1^ after 32 scans. The samples were placed directly on the ATR crystal.

### 3.3. Rheological Behavior

In order to analyze the gel-sol transition temperatures (T_gel-sol_) of the iongels, dynamic mechanical thermal analysis (DMTA) was performed using a parallel-plate geometry (8 mm in diameter), with a temperature sweep ranging from 20 to 120 °C and a heating rate of 2 °C min^−1^. The experiments were conducted at a frequency of 1.0 Hz and 0.1% of strain. An Anton Paar Physica MCR 301 rheometer was used to measure the rheological behavior of the polyphenol iongel materials.

### 3.4. Mechanical Properties

The mechanical properties were tested on a universal testing machine (INSTRON 3344) at 23 °C and 55% of relative humidity. Disks were prepared with around 1 mm in thickness and then subjected to compression, in which a 10 mm diameter plane-tip was moved down at a constant speed (1 mm·min^−1^) until compressing the samples 40% of their height. Five consecutive cycles were performed for each iongel sample.

### 3.5. Ionic Conductivity

The ionic conductivity of the polyphenol iongel was measured by electrochemical impedance spectroscopy (EIS) using an Autolab 302N potentiostat-galvanostat coupled to a Microcell HC station, with temperature control during the measurements. Circular samples of 8 mm in diameter were used. The samples were sandwiched between two stainless steel electrodes and sealed in the Microcell. The temperature was set between 20 to 60 °C with a step of 10 °C and 20 min of equilibration. Frequency ranged from 1.10–5 Hz to 1 Hz, and the employed amplitude was 10 mV.

## 4. Biological Assays of Iongel Materials

### 4.1. Cytotoxicity

To determine the possible cytotoxic effect of the polyphenol iongels, solutions of 100 µg/mL of PVA-[Ch][Van], PVA-[Ch][Sal], PVA-[Ch][Ga], and PVA-[Ch][Caff] were prepared in RPMI 1640 media without phenol red, and then, they were sterilized by filtration through Millipore filter membranes of 0.45 and 0.22 μm pore size. These solutions were kept sterile at 4 °C until their use.

Peritoneal macrophages were obtained by intraperitoneal puncture of CD1 mice (Mus musculus strain CD1), and the macrophage suspension was prepared in RPMI + fetal bovine serum 10% + antibiotics (Penicillin-Streptomycin 100,000 IU/L). The macrophage suspension was placed in a sterile conical tube to perform cell counting in a Neubauer chamber and kept at 4 °C until culture.

The animals were kept under the conditions recommended by animal welfare standards and after IACUC (Institutional Animal Care and Use Committee) approval CICUA-044-2021.

Briefly, 100 µL of 1 × 10^5^ cells were plated per well in a 96-well plate and left to stabilize for 2 hours before each experiment to allow cells to adhere to the plate surface. Cells were incubated at 37 °C in a humidified atmosphere of 95% of air and 5% of CO_2_.

Cell viability assay using XTT: To determine the cell viability of macrophages after exposure to each polyphenol iongel, cell viability was assessed using CyQuant XTT cell viability assay, which includes XTT reagent (2,3-Bis-(2-Methoxy-4-Nitro-5-Sulfophenyl)-2H-Tetrazolium-5-Carboxanilide) and an Electron Coupling Reagent. The XTT reagent, a tetrazolium-based compound, is sensitive to cellular redox potential, and therefore life cells reduce the compound and produce a colored formazan product that can be measured.

After 2 h of cell culture, 100 µL of each polyphenol iongel (100 µg/mL) was added to the cell culture and incubated for 20 h. Then, 100 µL of supernatant were carefully discarded and 70 µL of XTT reagent immediately prepared was added to each well and incubated for 4 h at 37 °C.

Finally, the absorbance (Abs) was measured at 450 nm using a 96-well plate reader, and the cell viability percentage was determined using a culture without any treatment or stimulus as control according to:

(1) $$\%\;\mathrm{cell}\;\mathrm{viability}=\frac{\mathrm{Abs}\;\mathrm{sample}}{\mathrm{Abs}\;\mathrm{control}\;}\times 100$$

Cell viability assay using vital dye: The macrophages were cultivated in microscope slides. Briefly, 300 µL of 1 × 10^5^ cells were seeded per slide and stabilized for 2 h at 37 °C in a humidified atmosphere of 95% of air and 5% of CO_2_ before the assay to allow their adhesion to the slide surface.

After the stabilization period, the macrophages culture and the polyphenol iongels were in contact for 24 h in the same incubation conditions, then the supernatant was removed, and phosphate buffer saline (PBS) was added to wash the slide. Immediately, 10 µL of the 0.4% trypan blue solution was added, and living and dead cells were counted under a microscope, expressing the viability in terms of percentage.

### 4.2. In Vitro Hemolysis and Agglutination Test

Serial dilutions from 100 µg/mL of each iongel were prepared and mixed with 50 µL of mice blood, previously mixed with EDTA as an anticoagulant, in a 96-well plate and incubated for 24 h at 4 °C. After this period, a qualitative analysis of lysis and agglutination was done in the stereoscope.

### 4.3. In Chemico Antioxidant Activity

The antioxidant activity (AA) of the polyphenol iongels was determined by a radical scavenging assay using DPPH. An aqueous solution of ascorbic acid (5.7 µmol/L) was used as a reference to compare the results because of its well-known antioxidant activity [25].

A DPPH solution of 0.5 µmol/L was prepared in methanol and added to 100 µL of 100 µg/mL solution of each polyphenol iongel; then, it was incubated for 120 min, and the absorbance at 515 nm was read with a 96-well microplate reader.

DPPH radical scavenging activity was determined according to:

(2) $$\%\mathrm{AA}=\frac{\left(\mathrm{Abs}\;\mathrm{control}-\mathrm{Abs}\;\mathrm{sample}\right)}{\mathrm{Abs}\;\mathrm{control}}\times 100$$

### 4.4. Anti-Inflammatory Assay

The potential anti-inflammatory activity of the polyphenol iongels was tested by analyzing their ability to inhibit or decrease nitric oxide production in LPS-stimulated macrophages using the Griess reagent. The Griess reaction is based on the formation of a chromophore by the reaction of sulfanilamide with nitrite in an acidic medium, followed by coupling with bicyclic amines such as N-1-(naphthyl) ethylenediamine dihydrochloride. Griess reagent was prepared by mixing a solution of N-1-(naphthyl) ethylenediamine dihydrochloride (0.1% *w*/*v* in 5% H_3_PO_4_) with a solution of sulfanilamide (1% *w*/*v* in 5% *w*/*v* of H_3_PO_4_). Parthenolide (10 mM) was used as a positive control because of its anti-inflammatory activity.

After stabilizing the cell culture for 2 h, different stimuli were added to the wells: 75 µL of 1 µg/mL LPS, 25 µL of 50, 100, and 200 µg/mL of each gel, and a mixture of LPS/iongels to evaluate if the phenolic-based materials reduce the nitric oxide produced by the LPS stimulus after 24 h. The colorimetric reaction was obtained by mixing 50 μL of the supernatant, and 100 μL of the Griess reagent, allowing the reaction to take place for 30 min in the dark at room temperature. A nitrite solution of known concentration was used as a standard, and a calibration curve was prepared in the corresponding range according to the behavior of the samples. Then, the absorbance was measured at 540 nm and reported as NO% of the control, where the control is the cell culture without stimulus.

(3) $$\mathrm{NO}\%\;\mathrm{of}\;\mathrm{control}=\frac{\mathrm{Concentation}\;\mathrm{of}\;\mathrm{sample}}{\mathrm{Concentration}\;\mathrm{of}\;\mathrm{control}}\times 100$$

### 4.5. Antibacterial Activity

The antibacterial activity of the iongels was evaluated using a modification of the Kirby–Bauer disk diffusion susceptibility test. A bacterial suspension of *Escherichia coli* was prepared using a 0.5 McFarland standard in sterile saline. Mueller–Hinton plates were previously prepared and then inoculated with the bacterial suspension using a sterile swab by streaking the swab three times over the entire agar surface.

Then, the iongel disks (6 mm) were placed on the agar surface and incubated for 18 h at 35 °C ± 2 °C. After this period of time, the inhibition zone was measured from edge to edge across the zone of inhibition over the center of the disk.

## 5. Results and Discussion

### 5.1. Preparation and Characterization of Iongel Materials

**Figure 1:**
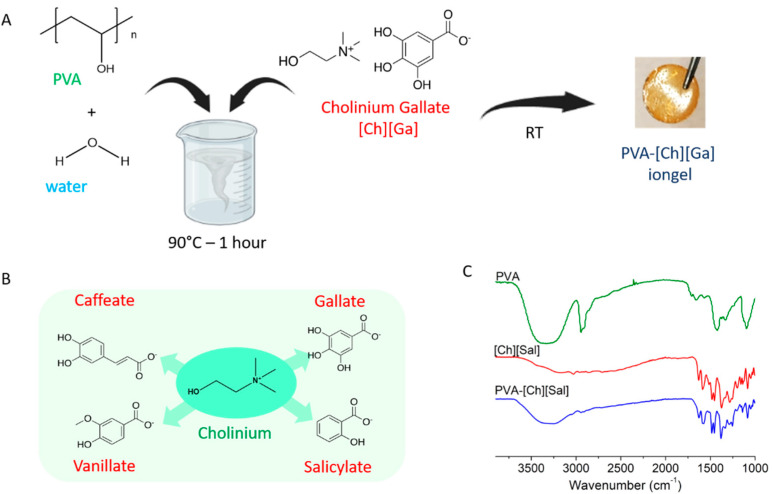
(**A**) Schematic representation of iongel formation by self-assembly of phenolic-based ILs and PVA and (**B**) chemical structures of the four phenolic-based ILs. (**C**) FTIR spectra for PVA, [Ch][Sal] IL, and PVA-[Ch][Sal] iongel.

The iongels were prepared using a simple hot dissolution/cooling self-assembly procedure. Using this method, soft solid iongel materials can be obtained in a fast way. Table S1 of the Supporting information (SI) summarizes the different polyphenol iongels obtained, while Figure 1A shows a schematic representation of the PVA-[Ch][Ga] iongel. The chemical structures of the investigated phenolic-based ILs are shown in Figure 1B. Both components of the iongel (IL and PVA) were combined to prepare iongels with 5%, 10%, and 20% of polymer PVA concentrations. In the case of PVA-[Ch][Ga], only iongels with 10% polymer concentration were obtained, while with 5% did not occur, and for 20%, a non-homogenous mixture was obtained. Therefore, the four polyphenol iongels with a polymer concentration of 10% were fully characterized.

ATR-FTIR was used to give more information about the interactions in the polymer network of the polyphenol iongels. Figure S1 shows the ATR-FTIR spectra of neat PVA, the four ILs, and the polyphenol iongels. In particular, Figure 1C displays the FTIR spectra of neat PVA, [Ch][Gal] IL, and PVA-[Ch][Gal] iongel, and it can be observed that the PVA and [Ch][Ga] ATR-FTIR spectra obtained are in agreement with those previously reported [13,14,26]. In the case of neat PVA, the peaks observed at 3014–3680 cm^−1^ can be attributed to the stretching vibration of OH groups. In addition, there are two bands, one at 2931 cm^−1^ due to the asymmetrical stretching of -CH groups and another at 2853 cm^−1^ related to the -CH symmetrical stretching.

Regarding the [Ch][Ga] IL, the band of the OH stretching appears at 3080 cm^−1^and the one related to the C=O stretching vibration can be seen at 1600 cm^−1^. On the other hand, the C-OH stretching vibrations, which are typical of phenol groups, appear in the region between 1200 and 1300 cm^−1^. A peak corresponding to the CN vibration from the cholinium group can also be observed at 1185 cm^−1^. Other characteristic signals of the IL can be found between 1100 and 750 cm^−1^, which correspond to the aromatic rings’ -CH out-of-plane bending vibrations. Although it is well known that the shift in the signal corresponding to the C=O stretching from 1710 to 1686 cm^−1^ is due to the formation of H bonding in PVA-phenolic-based hydrogel, no significant shifts in these bands were detected in the case of PVA-[Ch][Ga] iongel [11].

The thermal characteristics of polyphenol iongels were analyzed by TGA (Figure S2, SI). According to the decomposition pattern, the iongels displayed good thermal stability, with maximum decomposition temperatures (T_max_) between 206 and 242 °C. Additionally, the TGA analysis showed that the iongels bearing the [Ch][Van] IL showed the lowest degradation temperatures at 50% of weight loss (T_50%_), while the ones containing the [Ch][Caff] IL presented the highest stability (T_50%_ ≈ 275°C). These results are consistent with the neat ILs’ decomposition profiles shown in Figure S2 and Table S2 of SI.

**Figure 2:**
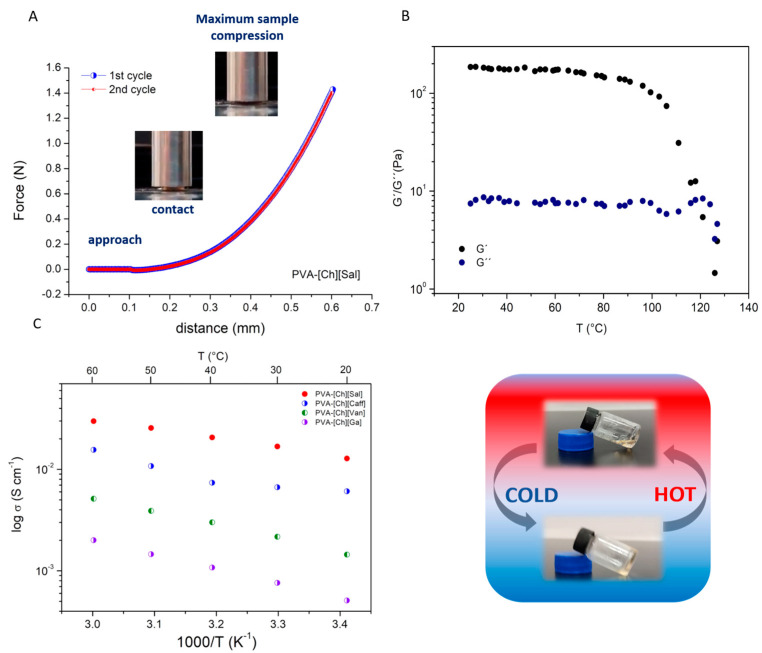
(**A**) Force vs. distance during two consecutive cycles of compression measured for the PVA-[Ch][Sal] iongel (10% polymer concentration). (**B**) Thermomechanical behavior of the PVA-[Ch][Sal] iongel and picture of the iongel transition (10% polymer concentration). (**C**) Ionic conductivity of phenolic-based iongels (10% of polymer concentration).

In wound healing applications, the mechanical and rheological properties of the materials are important aspects to be considered. The materials must be particularly soft, elastic, and flexible to form mechanically compliant interfaces with the skin. Figure 2A shows two typical consecutive compression curves for the axial compression until 40% deformation of the PVA-[Ch][Sal] iongels with 10% polymer concentration. The different cycles performed for each iongel started by the probe contacting the sample at the same point. In all cases, the iongels returned to their initial dimension after being compressed, as seen in Figure 2A. In summary, the material exhibits an elastic behavior, can resist high deformations, and recover its initial shape after load removal during consecutive cycles. All the polyphenol iongel materials revealed similar performances, which agrees with previous reports of similar systems [13,14,15].

Additionally, the rheological properties of the polyphenol iongels were evaluated using DMTA. As an example, Figure 2B shows the viscoelastic behavior of the PVA-[Ch][Sal] iongel as a function of the temperature. At temperatures below 120 °C, the elastic modulus (G′) is greater than the viscous modulus (G′′), indicating that polyphenol iongels exhibit solid-like characteristics. When the temperature increases, a transition from an elastic network to a viscoelastic liquid (G′′ > G′) occurs. The sol-gel transition temperature (T_gel-sol_, determined as the temperature at which G′′ = G′) for the polyphenol iongels ranged from 78 to 123 °C (Table S3 of SI). A reversible transition confirms the formation of the iongel structure by H-bonding, which makes this material very interesting to be processed by 3D printing.

Several authors have recently reported an acceleration in the regeneration tissue rate when low currents are applied to the wound [32,33]. Considering the potential of these iongels for electrically-stimulated wound healing, their ionic conductivity was measured (Figure 2C) with obtained values ranging from 1.2 × 10^−2^ and 7.4 × 10^−4^ S cm^−1^ at room temperature. These high values of ionic conductivity, which are in agreement to what has been reported for other iongel systems, [34] indicate that this property could add a further stimulus for wound healing to those provided by the bio-functionality of the iongels components (analyzed below).

### 5.2. Biological Activity

Different assays were carried out to determine the viability of the polyphenol iongels for wound healing applications. Firstly, the cytotoxicity of the iongels was tested on peritoneal macrophages from *Mus musculus* CD1, because of their significant role in the immune response [35]. Additionally, in vitro hemolysis and agglutination tests were carried out with blood from the same mice to evaluate the action of the iongels in erythrocytes as an important effect on wound healing treatments.

**Figure 3:**
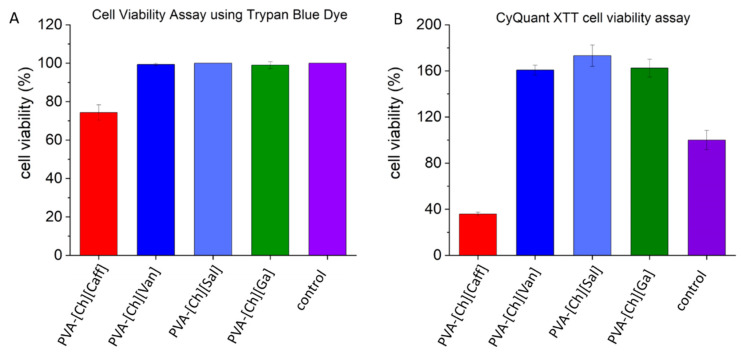
(**A**) Cell viability percentage after 24 h of exposure by trypan blue assay. (**B**) CyQuant XTT Cell viability after 24 h of exposure.

As evidenced in Figure 3A, the PVA-[Ch][Van], PVA-[Ch][Sal], and PVA-[Ch][Ga] samples have viability percentages of 99%, 100%, and 90%, respectively, behaving very similar to the control without any stimulus. The PVA-[Ch][Caff] sample showed the lowest percentage of cell viability at around 74%.

Cell viability was also determined using the CyQUANT™ XTT Cell Viability Assay, which is suggested for detecting mammalian cell viability [36,37]. As shown in Figure 3B, the PVA-[Ch][Caff] sample showed again the lowest percentage of cell viability 35 ± 0.01%, with 65% more cell mortality than the control. Moreover, in the case of PVA-[Ch][Van], PVA-[Ch][Sal], and PVA-[Ch][Ga] samples, they presented 161 ± 0.04%, 173 ± 0.09% and 162 ± 0.08%, respectively, of cell viability analyzed by this method.

The cell viability percentages above 100% in the CyQUANT™ XTT Cell Viability Assay may be due to three main reasons. The first is cell proliferation, which does not apply in this case because the primary culture used for peritoneal macrophages does not have the potential for proliferation; therefore, this factor is excluded. The second factor is due to the redox potential of the iongels that can reduce the reagent too, but based on the absorbance of the iongels alone with XTT reagent, no significant results were obtained; then, this is not the reason either [38]. Finally, the third possibility is that iongels stimulate macrophages and induce the production of ROS (reactive oxygen species) as a defense mechanism, which, based on the principle of the method used, can interfere in the reduction of the XTT used in this assay. However, this ROS production in a controlled way could have a great potential for wound healing applications because of the immune response stimulation [35,39]. In order to confirm if this stimulation and ROS production caused any damage, these results were correlated with those of cell viability with trypan blue, which do not suggest cell damage.

**Figure 4:**
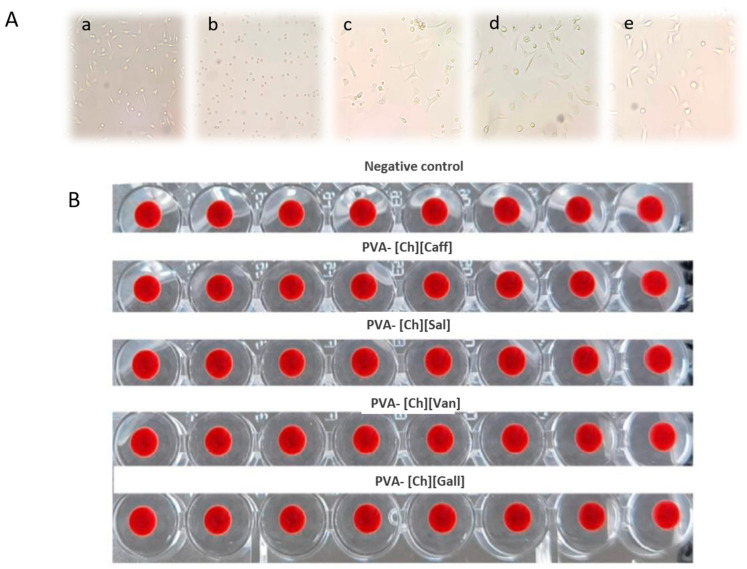
Qualitative results of cell culture and hemolysis test (**A**) Cell culture micrography after 24 h of incubation. (**a**) Control, (**b**) [Ch][Caff], (**c**) [Ch][Van], (**d**) [Ch][Sal], (**e**) [Ch][Ga]. (**B**) Hemolysis and agglutination test using mice blood after 24 h.

Additionally, as shown in Figure 4A, the cultures of the PVA-[Ch][Van], PVA-[Ch][Sal], and PVA-[Ch][Ga] samples under the microscope evidence viable cells adapted to cell culture with more elongated morphology unlike the culture exposed to PVA-[Ch][Caff] iongels, where only smaller and rounded cells are evidenced. This result correlates with the viability percentage obtained by both methods by suggesting that the increase in the percentage of cell viability may be due to cellular stimulation.

Regarding PVA-[Ch][Caff], which showed the lowest cell viability when evaluated by both techniques (Trypan blue and XTT) and different cell morphology, Chen et al. described that caffeate derivates have cytotoxic and apoptotic effects and can cause loss of mitochondria membrane potential [40].

This modification in the mitochondria is evidenced as low cell viability because the principle of the XTT assay is to evaluate cell viability through cellular respiration given in the mitochondria, and if there is damage in the membrane and morphology of the cell, the trypan blue dye can get into the cytoplasm confirming cell death.

Moreover, the in vitro hemolysis and agglutination tests help to identify if the iongels present hemolytic properties because they can harm patients’ health and interfere the wound healing process. As it can be observed in Figure 4B, there is no hemolytic effect and agglutination after 24 h of exposure of mice blood to each polyphenol iongel, which was repeated eight times with each material [41].

**Figure 5:**
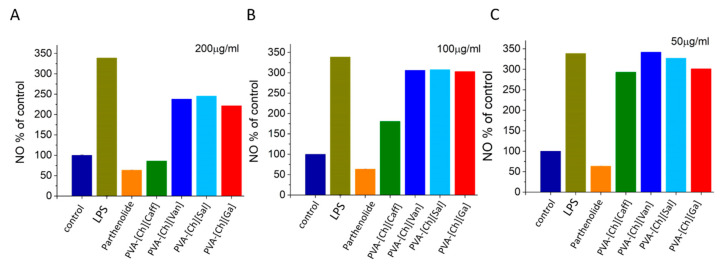
Evaluation of the capacity of iongels to prevent LPS-induced NO production in murine peritoneal macrophages different concentrations (200 µg/mL (**A**) 100 µg/mL (**B**) and 50 µg/mL (**C**)) of each polyphenol iongel reported as NO% of control.

One of the most interesting properties of these polyphenol iongels are their potential anti-inflammatory properties due to the presence of the phenolic IL anions. The nitric oxide production was assessed via a Griess assay [26,42]. Data are presented as NO% of control (Figure 5) based on a calibration curve using a nitrite standard and as the concentration of NO (Figure S3 of SI) [43]. The assay allowed us to quantify if there was a decrease in NO production between cells treated with LPS and cells treated with LPS in the presence of PVA-[Ch][Van], PVA-[Ch][Sal], PVA-[Ch][Ga], and PVA-[Ch][Caff] materials. As shown in Figure 5, the polyphenol iongels decreased the LPS-induced NO production, indicating the anti-inflammatory activity of the materials. There is an evident inhibition when higher concentrations are used (≤100 µg/mL).

The PVA-[Ch][Caff] sample (200 µg/mL) showed the best anti-inflammatory activity by reducing the NO production more than 63%, as the positive control of anti-inflammatory activity used (parthenolide). The PVA-[Ch][Van], PVA-[Ch][Sal], and PVA-[Ch][Ga] iongels diminish in 30%, 27%, and 35%, respectively, the NO production at the same concentration. At lower concentrations (50 and 100 µg/mL), the inhibition percentage is lower, but there is always an inhibition when compared with the culture with LPS stimulus (Figure 5). These results demonstrate better anti-inflammatory activity than those reported for nanostructured cellulose membranes loaded with cholinium-based ILs bearing caffeate and gallate anions [26].

**Figure 6:**
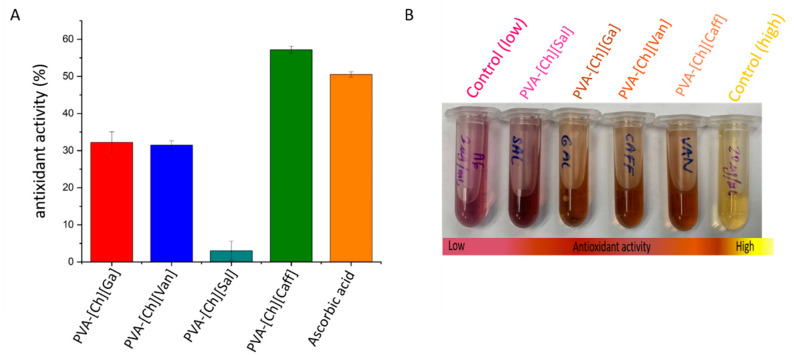
(**A**) Antioxidant activity of iongels quantified as the inhibition percentage of the DPPH radical via in chemico at 200 µg/µL. (**B**) Picture showing the color change of each polyphenol iongel and controls with DPPH reagent after 120 min.

Another key property of polyphenol iongels is their potential antioxidant activity. This property depends on the bioactive phenolic compounds, mainly due to the presence of pyrogallol and catechol groups, which can act as radical scavengers through an electron donation or hydrogen donation mechanism. Antioxidants can react with a stable radical (DPPH) by providing an electron or a hydrogen atom, thus reducing it to 2,2-diphenyl-1-hydrazine (DPPH-H) or analogous substituted hydrazine (DPPH-R) characterized by a pale yellow color that could be easily monitored at 515 nm [26,44]. Antioxidant activity, using DPPH reagent (a widely used technique to evaluate the ability of compounds to operate as free radical scavengers and hydrogen suppliers) is presented as percentage in Figure 6. As a reference, the ascorbic acid solution tested revealed 51% of antioxidant activity [45,46].

The antioxidant activities of the prepared polyphenol iongels varied between 3% to 57%, and the PVA-[Ch][Caff] material showed the maximum antioxidant capacity (57% ± 0.009 and inhibition of the DPPH radical), more than ascorbic acid, followed by PVA-[Ch][Ga] (32% ± 0.029), PVA-[Ch][Van] (31% ± 0.011), and PVA-[Ch][Sal] (3% ± 0.026). These results are in agreement to what was previously reported for the neat ILs [31].

Due to the well know antioxidant properties of polyphenols, different efforts to develop biomaterials employing these compounds have been carried out. For example, Amain et al. have used polyphenols in polymer networks as antidiabetic agents to improve human health care. In this way, the results obtained in our research show that the synthesized iongels have the potential as antioxidant materials, which could be used for different biomedical applications, including wound healing and dermal treatments [47]. Analyzing the tendency and variability between the iongels, the lowest antioxidant activity is for [Ch][Sal], the relation between this reduction and its chemical structure could be due to the presence of other molecules that can be interfering in the reaction because of the chelation of metals by the hydroxyl groups [48]. In salicylates, the possible interactions with non-redox active metals such as calcium or magnesium have been reported and both are components of the culture media used (RPMI) in the biological assays. Studies by Zhao et al. conclude that calcium affects the antioxidant activity of simple phenols extracted from plants, such as hydroxybenzoates [49,50]. The chelation of the calcium ion could explain the decrease in the antioxidant activity of PVA-[Ch][Sal], which has the lowest antioxidant activity compared to the rest of the gels. However, given the potential of these materials for biological applications, this interference should always be considered because of the presence of these metals in blood, sweat, and other biological fluids that may be present in dermal wounds.

Based on the potential use of the proposed polyphenol iongels for wound healing applications and considering the previous reports of the antimicrobial properties of the ILs against antibiotic-resistant pathogens [51], the antibacterial activity of synthesized iongels was also tested. Regarding the antibacterial activity of ILs, Nickfarjam et al. reported the antibacterial properties of different ILs and they observed that this property could be attributed to the adsorption of the IL by the cell membrane through electrostatic interactions, and because of both, penetration of the IL that may produce leakage of the cytoplasm and cell lysis [51]. Furthermore, Ibsen et al. mentioned that ionic liquids have great antibacterial properties that have been previously demonstrated [52].

**Figure 7:**
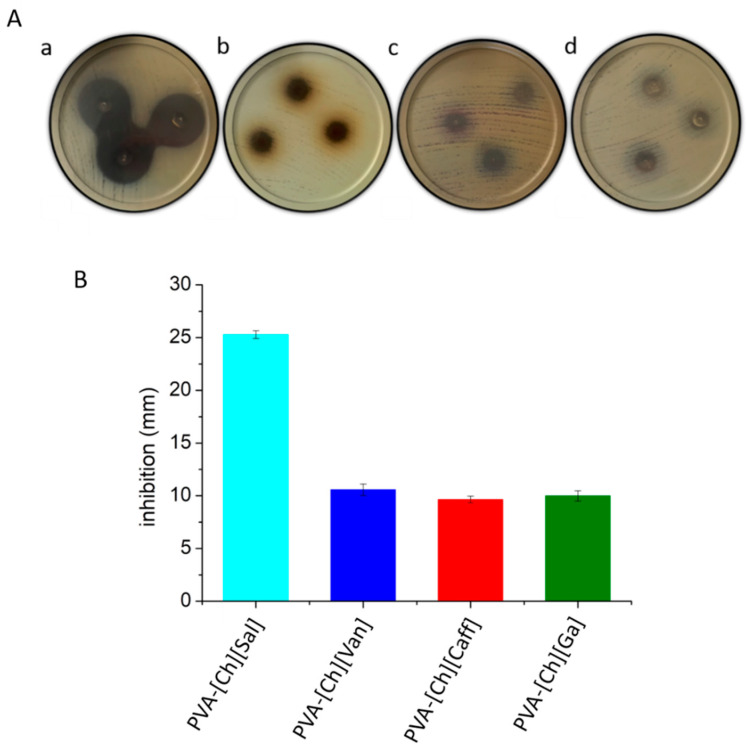
(**A**) Pictures of the inhibition of iongels using *Escherichia coli* after 18 h of incubation. (**a**) PVA-[Ch][Sal], (**b**) PVA-[Ch][Caff], (**c**) PVA-[Ch][Van], (**d**) PVA-[Ch][Ga]. (**B**) Antibacterial activity of iongels against *Escherichia coli*.

In order to determine if *Escherichia coli* is sensible to the polyphenol iongels, inhibition halos were compared with the standard pattern obtained with Gentamicin 10 µg/mL, which was reported as sensible with a halo ≥ 15 mm [53]. As shown in Figure 7A,B, the PVA-[Ch][Sal] presented the highest inhibitory activity (25 mm of inhibition), while the PVA-[Ch][Caff] had the lowest. Based on the reference of Gentamicine, we can conclude that *Escherichia coli* is more sensible to the PVA-[Ch][Sal] iongel. The other three iongels also show antibacterial activity but at a lower capacity when compared to that of the PVA-[Ch][Sal].

The highest antibacterial activity of PVA-[Ch][Sal] could be because salicylate and related compounds affect virulence factor production in some bacteria and are well-known as natural and safe antimicrobial agents. One of the mechanisms described for salicylic acid against *Escherichia coli* is the leakage of intracellular alkaline phosphatases and macromolecular substances (nucleic acids and proteins), which suggests the disruption of the bacterial cell wall [54].

## 6. Conclusions

In this work, we present new polyphenol iongel materials bearing PVA and phenolic-based ionic liquids. The iongels were prepared by an easy self-assembly process due to the H-bond interactions between the phenolic IL anion compounds and the PVA polymer matrix. Within the iongels, the phenolic IL anions play a dual role, acting as a crosslinker and as bioactive compounds. These iongels are flexible, elastic, and thermoreversible materials. Moreover, iongels with 10% polymer concentration demonstrated high biocompatibility, non-hemolytic activity, and non-agglutination in mice blood, which are key-sought specifications for materials in wound healing applications. All the iongels showed antibacterial properties, and PVA-[Ch][Sal] showed the highest inhibition halo for *Escherichia coli*. The polyphenol iongels also revealed high values of antioxidant activity, with the PVA-[Ch][Van] showing the highest activity. Regarding the anti-inflammatory activity, the four iongels showed a decrease in NO production in LPS-stimulated macrophages, with the PVA-[Ch][Sal] unveiling the best anti-inflammatory activity of more than 63% at 200 µg/mL.

## Data Availability

The data presented in this study are available on request from the corresponding author.

## Supplementary Materials

The following supporting information can be downloaded at https://www.mdpi.com/article/10.3390/polym15051076/s1. Table S1. Pictures of polyphenol iongels, Figure S1. FTIR spectra of neat PVA, ILs, and polyphenol iongels, Figure S2. TGA analysis of polyphenol iongels with 10% of polymer concentration and the corresponding ILs, Table S2 T_max_ and T50% of the polyphenol iongels, Table S3. Gel to sol transition temperatures of the polyphenol iongels, Figure S3. Evaluation of the capacity of iongels to prevent LPS-induced NO production in murine peritoneal macrophages different concentrations of each iongel reported NO (µg/mL).

## References

[1] Kost B., Brzezinski M., Socka M., Basko M., Biela T. (2020). Biocompatible Polymers Combined with Cyclodextrins: Fascinating Materials for Drug Delivery Applications. Molecules.

[2] Birajdar M.S., Joo H., Koh W.G., Park H. (2021). Natural Bio-Based Monomers for Biomedical Applications: A Review. Biomater. Res..

[3] Suarato G., Bertorelli R., Athanassiou A. (2018). Borrowing from Nature: Biopolymers and Biocomposites as Smart Wound Care Materials. Front. Bioeng. Biotechnol..

[4] Qi X., Xiang Y., Cai E., You S., Gao T., Lan Y., Deng H., Li Z.P., Hu R., Shen J. (2022). All-in-One: Harnessing Multifunctional Injectable Natural Hydrogels for Ordered Therapy of Bacteria-Infected Diabetic Wounds. Chem. Eng. J..

[5] Tejiram S., Kavalukas S.L., Shupp J.W., Barbul A. (2016). Wound Healing.

[6] Adomėnienė A., Venskutonis P.R. (2022). *Dioscorea* Spp.: Comprehensive Review of Antioxidant Properties and Their Relation to Phytochemicals and Health Benefits. Molecules.

[7] Guo S., DiPietro L.A. (2010). Critical Review in Oral Biology & Medicine: Factors Affecting Wound Healing. J. Dent. Res..

[8] Hupkens P., Boxma H., Dokter J. (1995). Tannic Acid as a Topical Agent in Burns: Historical Considerations and Implications for New Developments. Burns.

[9] Andrade R.G., Dalvi L.T., Silva J.M.C., Lopes G.K.B., Alonso A., Hermes-Lima M. (2005). The Antioxidant Effect of Tannic Acid on the in Vitro Copper-Mediated Formation of Free Radicals. Arch. Biochem. Biophys..

[10] Ninan N., Forget A., Shastri V.P., Voelcker N.H., Blencowe A. (2016). Antibacterial and Anti-Inflammatory PH-Responsive Tannic Acid-Carboxylated Agarose Composite Hydrogels for Wound Healing. ACS Appl. Mater. Interfaces.

[11] Euti E.M., Wolfel A., Picchio M.L., Romero M.R., Martinelli M., Minari R.J., Igarzabal C.I.A. (2019). Controlled Thermoreversible Formation of Supramolecular Hydrogels Based on Poly(Vinyl Alcohol) and Natural Phenolic Compounds. Macromol. Rapid Commun..

[12] Cencha L.G., Allasia M., Passeggi M.C.G., Gugliotta L.M., Minari R.J. (2021). Formulation of Self-Crosslinkable Hybrid Acrylic/Casein Latex by Tannic Acid. Prog. Org. Coat..

[13] Luque G.C., Picchio M.L., Martins A.P.S., Dominguez-Alfaro A., Tomé L.C., Mecerreyes D., Minari R.J. (2020). Elastic and Thermoreversible Iongels by Supramolecular PVA/Phenol Interactions. Macromol. Biosci..

[14] Luque G.C., Picchio M.L., Martins A.P.S., Dominguez-Alfaro A., Ramos N., del Agua I., Marchiori B., Mecerreyes D., Minari R.J., Tomé L.C. (2021). 3D Printable and Biocompatible Iongels for Body Sensor Applications. Adv. Electron. Mater..

[15] Aguzin A., Luque G.C., Ronco L.I., Del Agua I., Guzmán-González G., Marchiori B., Gugliotta A., Tomé L.C., Gugliotta L.M., Mecerreyes D. (2022). Gelatin and Tannic Acid Based Iongels for Muscle Activity Recording and Stimulation Electrodes. ACS Biomater. Sci. Eng..

[16] Shekaari H., Zafarani-Moattar M.T., Mokhtarpour M. (2022). Therapeutic Deep Eutectic Solvent-Based Ion-Gel as a Neoteric Drug Delivery Carrier for 5-Fluorouracil. J. Iran. Chem. Soc..

[17] Qader I.B., Prasad K. (2022). Recent Developments on Ionic Liquids and Deep Eutectic Solvents for Drug Delivery Applications. Pharm. Res..

[18] Mokhtarpour M., Shekaari H., Shayanfar A. (2020). Design and Characterization of Ascorbic Acid Based Therapeutic Deep Eutectic Solvent as a New Ion-Gel for Delivery of Sunitinib Malate. J. Drug Deliv. Sci. Technol..

[19] Joshi N., Rawat K., Solanki P.R., Bohidar H.B. (2015). Biocompatible Laponite Ionogels Based Non-Enzymatic Oxalic Acid Sensor. Sens. Bio-Sens. Res..

[20] De Anastro A.F., Porcarelli L., Hilder M., Berlanga C., Galceran M., Howlett P., Forsyth M., Mecerreyes D. (2019). UV-Cross-Linked Ionogels for All-Solid-State Rechargeable Sodium Batteries. ACS Appl. Energy Mater..

[21] Forsyth M., Porcarelli L., Wang X., Goujon N., Mecerreyes D. (2019). Innovative Electrolytes Based on Ionic Liquids and Polymers for Next-Generation Solid-State Batteries. Acc. Chem. Res..

[22] Sato T., Banno K., Maruo T., Nozu R. (2005). New Design for a Safe Lithium-Ion Gel Polymer Battery. J. Power Sources.

[23] Qin H., Owyeung R.E., Sonkusale S.R., Panzer M.J. (2019). Highly Stretchable and Nonvolatile Gelatin-Supported Deep Eutectic Solvent Gel Electrolyte-Based Ionic Skins for Strain and Pressure Sensing. J. Mater. Chem. C.

[24] Leleux P., Johnson C., Strakosas X., Rivnay J., Hervé T., Owens R.M., Malliaras G.G. (2014). Ionic Liquid Gel-Assisted Electrodes for Long-Term Cutaneous Recordings. Adv. Healthc. Mater..

[25] Tomé Liliana C., Luca P., Jason B., Forsyth Maria M.D. (2021). Emerging Iongel Materials towards Applications in Energy and Bioelectronics. Mater. Horizons.

[26] Morais E.S., Silva N.H.C.S., Sintra T.E., Santos S.A.O., Neves B.M., Almeida I.F., Costa P.C., Correia-Sá I., Ventura S.P.M., Silvestre A.J.D. (2019). Anti-Inflammatory and Antioxidant Nanostructured Cellulose Membranes Loaded with Phenolic-Based Ionic Liquids for Cutaneous Application. Carbohydr. Polym..

[27] De Fernandes I.A.A., Maciel G.M., Ribeiro V.R., Rossetto R., Pedro A.C., Haminiuk C.W.I. (2021). The Role of Bacterial Cellulose Loaded with Plant Phenolics in Prevention of UV-Induced Skin Damage. Carbohydr. Polym. Technol. Appl..

[28] Gomes J.M., Silva S.S., Fernandes E.M., Lobo F.C.M., Martín-Pastor M., Taboada P., Reis R.L. (2022). Silk Fibroin/Cholinium Gallate-Based Architectures as Therapeutic Tools. Acta Biomater..

[29] You S., Huang Y., Mao R., Xiang Y., Cai E., Chen Y., Shen J., Dong W., Qi X. (2022). Together Is Better: Poly(Tannic Acid) Nanorods Functionalized Polysaccharide Hydrogels for Diabetic Wound Healing. Ind. Crops Prod..

[30] Fang H., Wang J., Li L., Xu L., Wu Y., Wang Y., Fei X., Tian J., Li Y. (2019). A Novel High-Strength Poly(Ionic Liquid)/PVA Hydrogel Dressing for Antibacterial Applications. Chem. Eng. J..

[31] Sintra T.E., Luís A., Rocha S.N., Ferreira A.I.M.C.L., Gonçalves F., Santos L.M.N.B.F., Neves B.M., Freire M.G., Ventura S.P.M., Coutinho J.A.P. (2015). Enhancing the Antioxidant Characteristics of Phenolic Acids by Their Conversion into Cholinium Salts. ACS Sustain. Chem. Eng..

[32] Kanaan A.F., Piedade A.P., de Sousa H.C., Dias A.M.A. (2021). Semi-Interpenetrating Chitosan/Ionic Liquid Polymer Networks as Electro-Responsive Biomaterials for Potential Wound Dressings and Iontophoretic Applications. Mater. Sci. Eng. C.

[33] Liu B., Fu R., Duan Z., Zhu C., Deng J., Fan D. (2022). Ionic Liquid-Based Non-Releasing Antibacterial, Anti-Inflammatory, High-Transparency Hydrogel Coupled with Electrical Stimulation for Infected Diabetic Wound Healing. Compos. Part B Eng..

[34] Gachuz E.J., Castillo-Santillán M., Juarez-Moreno K., Maya-Cornejo J., Martinez-Richa A., Andrio A., Compañ V., Mota-Morales J.D. (2020). Electrical Conductivity of an All-Natural and Biocompatible Semi-Interpenetrating Polymer Network Containing a Deep Eutectic Solvent. Green Chem..

[35] Toribio L.A., Doctoral T. (2008). Mecanismos Moleculares Que Regulan La Activación Clásica y Alternativa En Los Macrófagos.

[36] Thermo Fisher Scientific (2018). CyQUANT TM XTT Cell Viability Assay. www.thermofisher.com/us/en/home/global/terms-and-conditions.html.

[37] Abcam (2020). XTT Assay Kit (ab232856). https://www.abcam.com/ps/products/232/ab232856/documents/XTT-Assay-protocol-book-v1a-ab232856%20.

[38] Fischer J., Prosenc M.H., Wolff M., Hort N., Willumeit R., Feyerabend F. (2010). Interference of Magnesium Corrosion with Tetrazolium-Based Cytotoxicity Assays. Acta Biomater..

[39] Ding A.H., Nathan C.F., Stuehr D.J. (1988). Release of Reactive Nitrogen Intermediates and Reactive Oxygen Intermediates from Mouse Peritoneal Macrophages. Comparison of Activating Cytokines and Evidence for Independent Production. J. Immunol..

[40] Chen Y.C., Kuo Y.H., Yang N.C., Liu C.W., Chang W.T., Hsu C.L. (2014). Cytotoxic and Apoptotic Effects of Caffeate Derivatives on A549 Human Lung Carcinoma Cells. J. Chin. Med. Assoc..

[41] Von Petersdorff-Campen K., Daners M.S. (2022). Hemolysis Testing In Vitro: A Review of Challenges and Potential Improvements. ASAIO J..

[42] Wu C.H., Chen T.L., Chen T.G., Ho W.P., Chiu W.T., Chen R.M. (2003). Nitric Oxide Modulates Pro- and Anti-Inflammatory Cytokines in Lipopolysaccharide-Activated Macrophages. J. Trauma.

[43] Meng F., Lowell C.A. (1997). Lipopolysaccharide (LPS)-Induced Macrophage Activation and Signal Transduction in the Absence of Src-Family Kinases Hck, Fgr, and Lyn. J. Exp. Med..

[44] Pisoschi A.M., Pop A., Cimpeanu C., Predoi G. (2016). Antioxidant Capacity Determination in Plants and Plant-Derived Products: A Review. Oxid. Med. Cell. Longev..

[45] Garcia E.J., Cadorin Oldoni T.L., de Alencar S.M., Reis A., Loguercio A.D., Miranda Grande R.H. (2012). Antioxidant Activity by DPPH Assay of Potential Solutions to Be Applied on Bleached Teeth. Braz. Dent. J..

[46] Rahman M.M., Islam M.B., Biswas M., Khurshid Alam A.H.M. (2015). In Vitro Antioxidant and Free Radical Scavenging Activity of Different Parts of Tabebuia Pallida Growing in Bangladesh. BMC Res. Notes.

[47] Shavandi A., Bekhit A.E.D.A., Saeedi P., Izadifar Z., Bekhit A.A., Khademhosseini A. (2018). Polyphenol Uses in Biomaterials Engineering. Biomaterials.

[48] Kumar S., Sandhir R., Ojha S. (2014). Evaluation of Antioxidant Activity and Total Phenol in Different Varieties of Lantana Camara Leaves. BMC Res. Notes.

[49] Zhao Z., Vavrusova M., Skibsted L.H. (2018). Antioxidant Activity and Calcium Binding of Isomeric Hydroxybenzoates. J. Food Drug Anal..

[50] Baltazar T.M., Dinis-Oliveira J.R., Duarte A.J., Bastos L.M., Carvalho F. (2011). Antioxidant Properties and Associated Mechanisms of Salicylates. Curr. Med. Chem..

[51] Nikfarjam N., Ghomi M., Agarwal T., Hassanpour M., Sharifi E., Khorsandi D., Ali Khan M., Rossi F., Rossetti A., Nazarzadeh Zare E. (2021). Antimicrobial Ionic Liquid-Based Materials for Biomedical Applications. Adv. Funct. Mater..

[52] Ibsen K.N., Ma H., Banerjee A., Tanner E.E.L., Nangia S., Mitragotri S. (2018). Mechanism of Antibacterial Activity of Choline-Based Ionic Liquids (CAGE). ACS Biomater. Sci. Eng..

[53] Kuang X., Hao H., Dai M., Wang Y., Ahmad I., Liu Z., Yuan Z. (2015). Serotypes and Antimicrobial Susceptibility of Salmonella Spp. Isolated from Farm Animals in China. Front. Microbiol..

[54] Song X., Li R., Zhang Q., He S., Wang Y. (2022). Antibacterial Effect and Possible Mechanism of Salicylic Acid Microcapsules against Escherichia Coli and Staphylococcus Aureus. Int. J. Environ. Res. Public Health.

