# Supplementary material for: Polyphenol Iongel Patches with Antimicrobial, Antioxidant and Anti-Inflammatory Properties
Source: Polymers (Basel). 2023 Feb 21;15(5):1076. doi: 10.3390/polym15051076 (PMC10007217; doi:10.3390/polym15051076)
Supplement: Supplementary file 1 [file polymers-15-01076-s001.zip › polymers-2192887-supplementary.pdf]

Supporting Information

Polyphenol iongel patches with antimicrobial, antioxidant and anti-inflammatory properties

Gisela C. Luque[a]*, Melissa Moya[b,c], Matias Picchio[a], Vanessa Bagnarello[b,e], Idalia Valerio[b,c,d], José Bolaños[b], María Vethencourt[b], Sue Hellen Gamboa[c,d], Liliana C. Tomé[f], Roque J. Minari[a,i]*and David Mecerreyes[gh]*

[a]Instituto de Desarrollo Tecnológico para la Industria Química (INTEC), CONICET, Güemes 3450, Santa Fe 3000, Argentina.

[b]Laboratorio de Investigación, Universidad de Ciencias Médicas, San José, Costa Rica.

[c]Facultad de Microbiología, Universidad de Ciencias Médicas, San José, Costa Rica.

[d]Facultad de Medicina, Universidad de Ciencias Médicas, San José, Costa Rica.

[e]Escuela de Fisioterapia, Universidad de Ciencias Médicas, San José, Costa Rica.

[f] LAQV-REQUIMTE, Chemistry Department, NOVA School of Science and Technology, FCT NOVA, Universidade NOVA de Lisboa, 2829-516 Caparica, Portugal.

[g] Ikerbasque, Basque Foundation for Science, 48013 Bilbao, Spain.

[h] POLYMAT University of the Basque Country UPV/EHU, Joxe Mari Korta Center, Avda. Tolosa 72, 20018 Donostia-San Sebastian, Spain.

[i] Facultad de Ingeniería Química (Universidad Nacional del Litoral), Santiago del Estero 2829, Santa Fe 3000, Argentina.

*Corresponding authors:

David Mecerreyes (E-mail: david.mecerreyes@ehu.es)

Roque J. Minari (E-mail: rjminari@santafe-conicet.gov.ar)

Gisela Luque (E-mail: luquegc@intec.unl.edu.ar)

Table S1. Pictures of polyphenol iongels

iongel	[Ch][Sal]	[Ch][Van]	[Ch][Caff]	[Ch][Ga]
5%	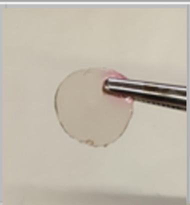	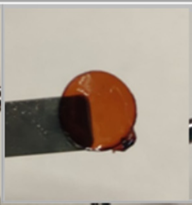	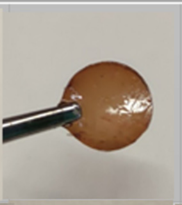	
10%	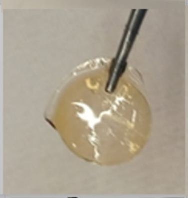	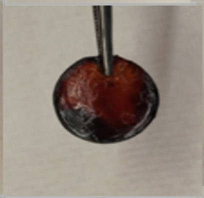	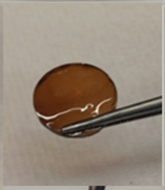	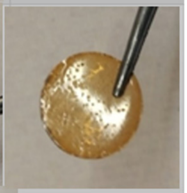
20%	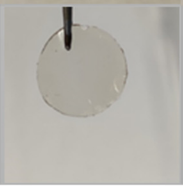	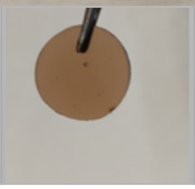	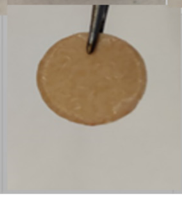	

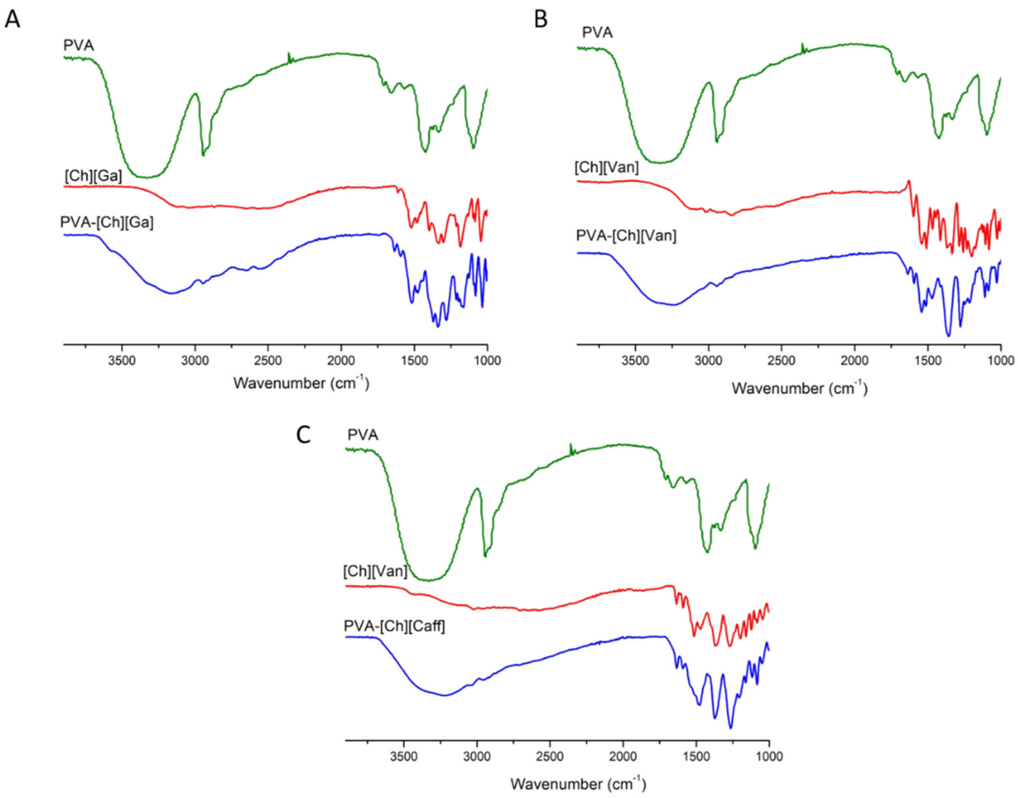

Figure S1. FTIR spectra of neat PVA, ILs, and polyphenol iongels.

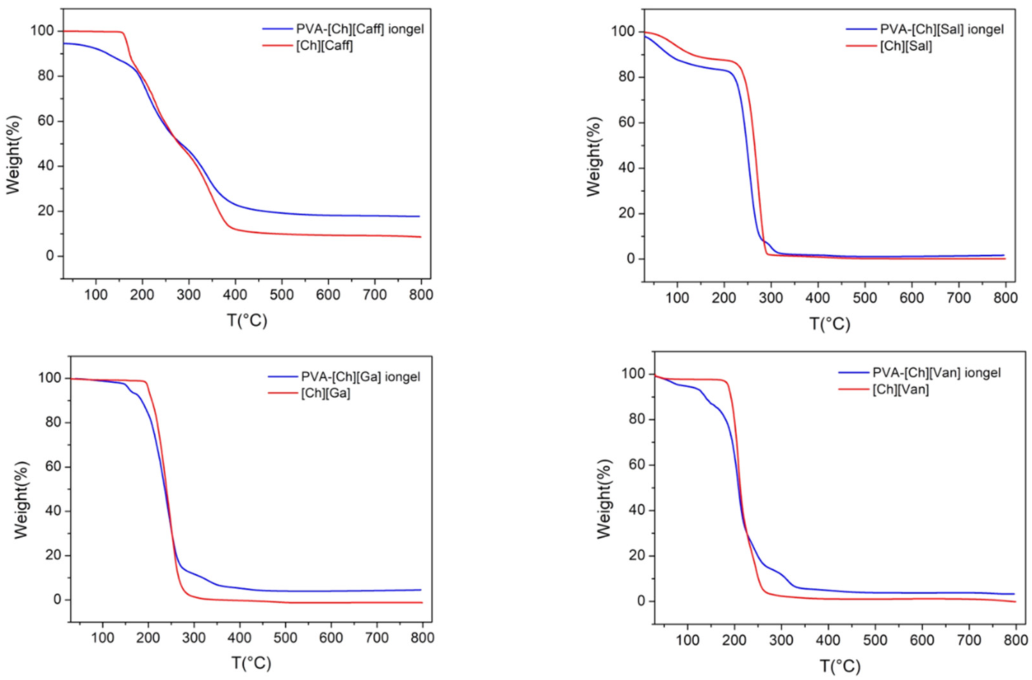

Figure S2. TGA analysis of polyphenol iongels with 10% of polymer concentration and the corresponding ILs.

Table S2 T_{max} and $T_{50\%}$ of the polyphenol iongels

**Iongel**	**T_{max} (°C)**	**$T_{50\%}$ (°C)**
PVA-[Ch][Ga]	242	241
PVA-[Ch][Van]	206	208
PVA-[Ch][Sal]	251	248
PVA-[Ch][Caff]	226	275

Table S3. Gel to sol transition temperatures of the polyphenol iongels.

Iongel	Transition temperatures (°C) $T_{gel\text{-}sol}$
PVA-[Ch][Ga]	123
PVA-[Ch][Van]	78
PVA-[Ch][Sal]	120
PVA-[Ch][Caff]	79

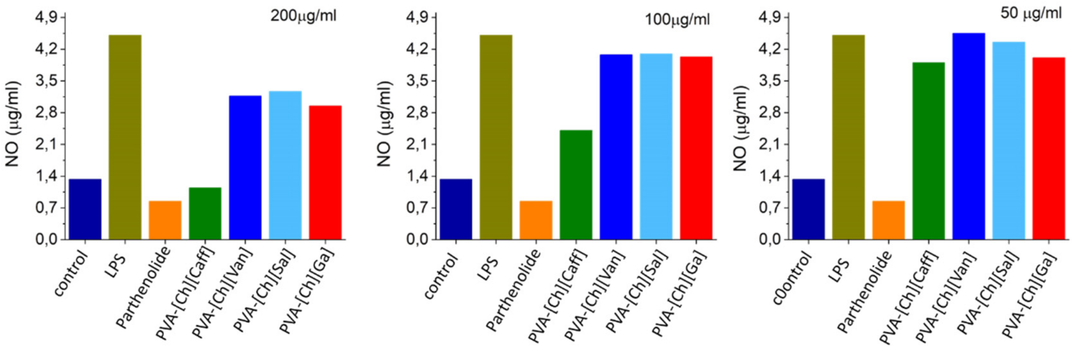

Figure S3. Evaluation of the capacity of iongels to prevent LPS-induced NO production in murine peritoneal macrophages different concentrations of each iongel reported NO (μg/ml)
